# Changes in Intrinsic Antibiotic Susceptibility during a Long-Term Evolution Experiment with Escherichia coli

**DOI:** 10.1128/mBio.00189-19

**Published:** 2019-03-05

**Authors:** Otmane Lamrabet, Mikaël Martin, Richard E. Lenski, Dominique Schneider

**Affiliations:** a Centre National de la Recherche Scientifique (CNRS), Grenoble Institut National Polytechnique (INP), Techniques de l’Ingénierie Médicale et de la Complexité–Informatique, Mathématiques et Applications, Grenoble, (TIMC-IMAG), Université Grenoble Alpes, Grenoble, France; b Department of Microbiology and Molecular Genetics, Michigan State University, East Lansing, Michigan, USA; University of British Columbia; Uppsala University; Technion

**Keywords:** *Escherichia coli*, antibiotic resistance, evolution, mutation accumulation, pleiotropy

## Abstract

High-level resistance often evolves when populations of bacteria are exposed to antibiotics, by either mutations or horizontally acquired genes. There is also variation in the intrinsic resistance levels of different bacterial strains and species that is not associated with any known history of exposure. In many cases, evolved resistance is costly to the bacteria, such that resistant types have lower fitness than their progenitors in the absence of antibiotics. Some longer-term studies have shown that bacteria often evolve compensatory changes that overcome these tradeoffs, but even those studies have typically lasted only a few hundred generations. In this study, we examine changes in the susceptibilities of 12 populations of Escherichia coli to 15 antibiotics after 2,000 and 50,000 generations without exposure to any antibiotic. On average, the evolved bacteria were more susceptible to most antibiotics than was their ancestor. The bacteria at 50,000 generations tended to be even more susceptible than after 2,000 generations, although most of the change occurred during the first 2,000 generations. Despite the general trend toward increased susceptibility, we saw diverse outcomes with different antibiotics. For streptomycin, which was the only drug to which the ancestral strain was highly resistant, none of the evolved lines showed any increased susceptibility. The independently evolved lineages often exhibited correlated responses to the antibiotics, with correlations usually corresponding to their modes of action. On balance, our study shows that bacteria with low levels of intrinsic resistance often evolve to become even more susceptible to antibiotics in the absence of corresponding selection.

## Abstract (precis)

Resistance to antibiotics often evolves when bacteria encounter antibiotics. However, bacterial strains and species without any known exposure to these drugs also vary in their intrinsic susceptibility. In many cases, evolved resistance has been shown to be costly to the bacteria, such that resistant types have reduced competitiveness relative to their sensitive progenitors in the absence of antibiotics. In this study, we examined changes in the susceptibilities of 12 populations of Escherichia coli to 15 antibiotics after 2,000 and 50,000 generations without exposure to any drug. The evolved bacteria tended to become more susceptible to most antibiotics, with most of the change occurring during the first 2,000 generations, when the bacteria were undergoing rapid adaptation to their experimental conditions. On balance, our findings indicate that bacteria with low levels of intrinsic resistance can, in the absence of relevant selection, become even more susceptible to antibiotics.

## INTRODUCTION

Resistance often evolves quickly when populations of bacteria are exposed to antibiotics, by either mutations or horizontally acquired resistance determinants (1, 2). In addition, there is variation in the intrinsic resistance of different bacterial strains and species to antibiotics that is not associated with any known history of exposure to these drugs (3). In many cases, evolved resistance has been shown to be costly to the bacteria, such that resistant genotypes have reduced growth and competitive fitness relative to their sensitive progenitors in the absence of antibiotics (4–9). However, studies have shown that bacteria often evolve compensatory changes that reduce or eliminate these tradeoffs (10–16). Even these studies, though, have typically run for only a few hundred bacterial generations.

In this study, we examine changes in antibiotic susceptibility during a long-term evolution experiment (LTEE) with Escherichia coli. In that experiment, 12 populations were founded from the same ancestral strain in 1988. Since then, they have been propagated in a defined carbon-limited medium without any exposure to antibiotics; the daily 100-fold dilutions and regrowth to stationary phase allow ∼6.7 generations per day (17–19). In this study, we measure the susceptibilities of the ancestral strain and evolved clones sampled from each population after 2,000 and 50,000 generations to the 15 antibiotics listed in Table 1. For 14 of these drugs, the ancestral strain has no known history of exposure; in the case of streptomycin, the ancestral strain was resistant, owing to a mutation in the *rpsL* gene that was intentionally selected many years before the long-term experiment began (20, 21).

**TABLE 1:** Antibiotics used in this study and their corresponding MIC values for the LTEE ancestral strain REL606

Antibiotic	Abbreviation^*a*^	Family	Cellular target	Ancestral MIC (μg/ml)
Amikacin	AMK	Aminoglycoside	Protein synthesis, binds 30S subunit	4
Amoxicillin-clavulanic acid	AMC	β-Lactam with β-lactamase inhibitor	Cell wall synthesis	1
Ampicillin	AMP	β-Lactam	Cell wall synthesis	2
Cefotaxime	CTX	β-Lactam	Cell wall synthesis	0.06
Ceftriaxone	CRO	β-Lactam	Cell wall synthesis	0.12
Chloramphenicol	CHL	Other	Protein synthesis, binds 50S subunit	1
Ciprofloxacin	CIP	Fluoroquinolone	DNA synthesis	0.005
Colistin	CST	Polypeptide	Cell membranes	0.125
Imipenem	IPM	β-Lactam	Cell wall synthesis	0.25
Levofloxacin	LVX	Fluoroquinolone	DNA synthesis	0.015
Rifampin	RIF	Polyketide	Transcription, binds β-subunit of RNA polymerase	16
Streptomycin	STR	Aminoglycoside	Protein synthesis, binds 30S subunit	128
Trimethoprim-sulfamethoxazole	SXT	Sulfonamide	Folate synthesis	0.25
Tetracycline	TET	Tetracycline	Protein synthesis, binds 30S subunit	0.5
Ticarcillin	TIC	β-Lactam	Cell wall synthesis	4

a Abbreviations recommended at https://aac.asm.org/content/abbreviations-and-conventions.

The central question that we address is whether the intrinsic ancestral resistance levels persist over evolutionary time. The evolved bacteria might become even more susceptible if the low-level intrinsic resistance of the ancestor to antibiotics imposed a cost in terms of their growth and competitiveness in the absence of drugs. Such low-level resistance might reflect a history of exposure to similar compounds produced by competitors or hosts (e.g., bile salts) in nature that might, without constant reinforcement, be diminished over time. In a similar vein, some unused catabolic functions have declined or been lost in the bacteria during the long-term evolution experiment (22–25). It is also possible, however, that increased resistance might evolve, even in the absence of exposure to antibiotics, as a correlated response to selection on other phenotypic traits. Indeed, Knöppel et al. (26) observed that a small percentage of experimental populations of E. coli and Salmonella enterica evolved reduced susceptibility to several antibiotics without any exposure. Similarly, some LTEE populations have evolved resistance to bacteriophage lambda, despite not encountering any viruses during the experiment (27). This surprising outcome resulted from the fact that, during their adaptation to a glucose-limited medium, the bacteria evolved reduced expression of LamB, a porin that facilitates their growth on maltose but which is also the receptor for lambda’s adsorption to the cell surface (24, 27). Moreover, many beneficial mutations during the LTEE affect global regulatory genes (28), thereby restructuring regulatory networks (29) and resulting in pleiotropic effects that could impact cellular responses to antibiotics.

## RESULTS

### Ancestral susceptibility profile.

Table 1 shows the MICs estimated for the LTEE ancestral strain for the 15 antibiotics used in this study. Each estimate is the median of the results from three replicate assays (see Data Set S1 in the supplemental material). For 12 of the antibiotics, the three replicate values were identical, whereas for 3 of them (amoxicillin-clavulanic acid [AMC], chloramphenicol [CHL], and rifampin [RIF]), one replicate assay deviated minimally (i.e., by a factor of 2), indicating the high repeatability of the measurements.

10.1128/mBio.00189-19.1 DATA SET S1 MIC values. Download Data Set S1, XLS file, 0.1 MB. Copyright © 2019 Lamrabet et al. 2019 Lamrabet et al. This content is distributed under the terms of the Creative Commons Attribution 4.0 International license.

### Susceptibility profiles of evolved clones.

We also estimated the MICs for the 24 evolved clones (from generations 2,000 and 50,000 for each of the 12 LTEE populations) for the same 15 antibiotics. As seen for the ancestor, the three replicate assays usually yielded identical MICs (Data Set S1). Across the 360 sets of replicates (24 clones × 15 antibiotics), the three assays gave identical MICs in 220 cases (61.1%), they deviated minimally (i.e., by a factor of 2) in 132 cases (36.7%), and in only 8 cases (2.2%) did they deviate more (in all of these cases by a factor of 4). The causes of the larger deviations are unknown. One possibility is that outliers result from resistant mutants that arise during the outgrowth of clonal isolates. If so, one would expect the outlier assays to have higher MICs than the two other assays for the same clone and antibiotic. However, the observed pattern was symmetrical, with the outliers having higher or lower MICs in four cases each. Subtle unintended variation in assay conditions is another potential source of experimental noise. In any case, large deviations were rare, and they serve to illustrate the value of performing replicate assays.

### Evolved clones tend to be more susceptible than the ancestor.

**FIG 1:**
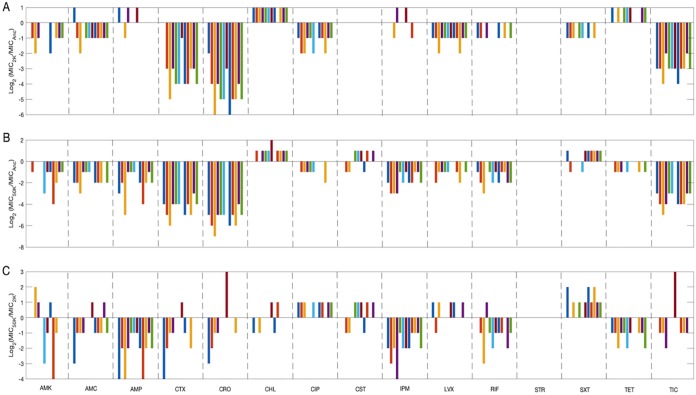
Changes in MIC values across generations. (A) Comparison of clones sampled at 2,000 generations (2K) and the ancestral strain (Anc). (B) Comparison of clones sampled at 50,000 generations (50K) and the ancestral strain. (C) Paired comparison of clones sampled at 50,000 and 2,000 generations from the same LTEE population. In all panels, each value is the log_2_-transformed ratio of the median MICs for the relevant strains.

Figure 1 shows the differences in antibiotic susceptibility between the ancestor and the evolved clones from generations 2,000 (Fig. 1A) and 50,000 (Fig. 1B), expressed as the log_2_-transformed ratio of their MICs. We characterized the overall pattern of changes in antibiotic susceptibility profiles in two different ways. We first asked how many of the 360 tests of the evolved clones had median MICs that were lower, the same as, or higher than the median MIC for the ancestor. The evolved MIC was lower than the ancestral value in 201 cases (55.8%), the same as the ancestral MIC in 114 cases (31.7%), and higher than the ancestor in 45 cases (12.5%). The excess of lower MICs (increased susceptibility) relative to higher MICs (increased resistance) in the evolved clones is highly significant relative to the null hypothesis of equally frequent changes in either direction (*P < *0.0001, one-tailed sign test).

We also performed a more stringent comparison based on only those cases where all three replicate assays of an evolved clone yielded MICs that were lower or higher than any of the three assays for the ancestor (Data Set S1). Using this criterion, there were 180 cases (50.0%) in which an evolved clone was uniformly more sensitive than the ancestor and only 18 cases (5.0%) in which an evolved clone was consistently more resistant than the ancestor; in the other 162 cases, there was overlap (including ties) between the evolved and ancestral assays. We can again confidently reject the null hypothesis that increased susceptibility and increased resistance are equally likely outcomes (*P < *0.0001, one-tailed sign test).

### Evolved clones from 50,000 generations tend to be more susceptible than those from 2,000 generations.

We asked next whether the tendency of the evolved clones to be more susceptible than the ancestor was more pronounced after 50,000 generations than at 2,000 generations. Using the stringent criterion of no overlap in the replicate MIC values obtained for a given evolved clone and the ancestor, there were 75 cases (41.7% of 180) of increased susceptibility in the 2,000-generation clones and 105 such cases (58.3% of 180) at 50,000 generations. These data thus support the hypothesis that the bacteria continued to become more susceptible over time (*P = *0.0011, one-tailed Fisher’s exact test).

While this temporal trend is significant, it is also important to emphasize that the changes are not proportional to the time scale. The mean change in susceptibility from the ancestor to the 2,000-generation clones (averaging the median log_2_-transformed changes over the 15 antibiotics and 12 populations) is –0.944 (Fig. 1A), corresponding to an almost 2-fold increase in average susceptibility. The mean change from generation 2,000 to 50,000 was –0.444 (Fig. 1C), which represents a further increase in average susceptibility of only about 36%, despite the 24-fold longer duration of the latter phase.

### Evolved clones from 50,000 generations tend to be more variable than those from 2,000 generations.

We also asked whether variation among the 12 independently derived clones in their MIC values tended to increase between 2,000 and 50,000 generations. (The LTEE populations were started from the same ancestor, and so the among-lineage variation could only increase from 0 to 2,000 generations.) To assess this variation, we computed the range in the log_2_-transformed median MIC values across the 12 clones at each time point for the 15 antibiotics tested. We then asked whether the ranges in median MICs tended to be greater at 50,000 generations than at 2,000 generations. For 10 antibiotics, the range was greater at 50,000 generations, while for the other 5 antibiotics, the ranges were the same at 2,000 and 50,000 generations. These data thus support the hypothesis that the LTEE lines became more heterogeneous in their susceptibility over time (*P = *0.0010, one-tailed sign test excluding the 5 ties).

### Variability in evolutionary trends among antibiotics.

Against these general tendencies for both the median susceptibility and the variability among the LTEE lineages to increase over time, we saw a range of different outcomes for the 15 different antibiotics. Here, we describe the specific patterns observed for the various antibiotics.

Streptomycin (STR) is the only antibiotic we tested to which the ancestral strain of the LTEE was resistant. Resistance to this drug was intentionally selected in the 1960s for use as a genetic marker in a mapping study (17, 20, 21, 30). Interestingly, streptomycin is also the only antibiotic for which none of the evolved LTEE lines showed any changes whatsoever in their MIC values. The *rpsL* mutation that confers streptomycin resistance was almost certainly costly (i.e., had reduced fitness in the absence of antibiotic) when it first arose, but the bacteria evidently acquired a compensatory mutation prior to the LTEE (11, 31). We consider the case of streptomycin resistance further in the Discussion.

For eight antibiotics (amikacin [AMK], AMC, cefotaxime [CTX], ceftriaxone [CRO], ciprofloxacin [CIP], levofloxacin [LVX], ticarcillin [TIC], and RIF), the median evolved clone sampled at both 2,000 and 50,000 generations was more susceptible than the ancestral strain. Even within these eight, however, there was considerable variation in the timing, magnitude, and variability of these changes. In the case of LVX, for example, the MICs of most of the evolved clones from both 2,000 and 50,000 generations differed from the ancestral MIC by only 2-fold. In some other cases, including AMC and RIF, susceptibility continued to increase gradually between 2,000 and 50,000 generations. For CRO and CTX, in contrast, the typical evolved clone from both generational samples was at least 16-fold more sensitive than the ancestor, and there were also 32-fold or even greater differences in MICs among the evolved clones. For two antibiotics (ampicillin [AMP] and imipenem [IPM]), increased susceptibility was typically only evident after 50,000 generations.

The other four antibiotics exhibited more idiosyncratic and unexpected patterns. In the case of CHL, the typical evolved clone from both generational samples appeared to be more resistant than the ancestor, although with only a minimal (i.e., 2-fold) increase in MIC. For trimethoprim-sulfamethoxazole (SXT), a typical clone from generation 2,000 was more sensitive than the ancestor, while most clones from generation 50,000 were more resistant. With tetracycline (TET), the opposite pattern held, with the earlier clones being more resistant than the ancestor and the later ones more sensitive. For both SXT and TET, the magnitude of these differences was again minimal (i.e., 2-fold) in each direction relative to the ancestor. Yet another pattern was evident for colistin (CST), in that there were no changes in any of the 2,000-generation clones, while some of the 50,000-generation clones were more sensitive than the ancestor and others more resistant.

### Evolutionary patterns in relation to modes of antibiotic action.

**FIG 2:**
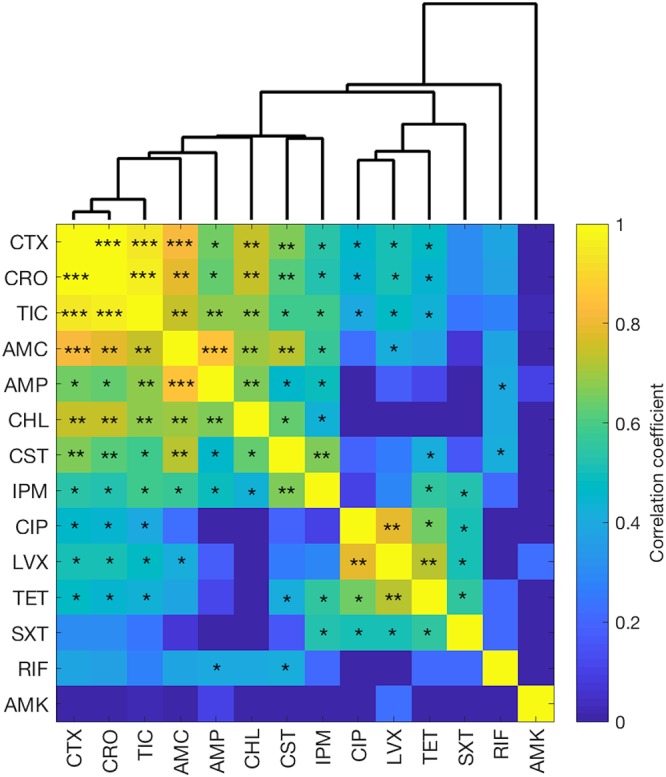
Correlated changes in antibiotic susceptibility. Correlation coefficients were calculated using log_2_-transformed ratios of the median MIC for each 50,000-generation clone and the common ancestor. The precise values of the coefficients are shown in Data Set S1, including occasional negative values that are colored as though they were zero. The matrix is symmetrical across the diagonal; the diagonal values are not meaningful. There were no changes in susceptibility to STR, which is not shown. Forty-nine of the 91 correlations are positive and significant at a *P *value of <0.1. ***, *P < *0.001; **, 0.001 < *P < *0.01; *, 0.01 < *P < *0.1. The dendrogram shows hierarchical clustering based on nearest neighbors.

We examined the correlated changes in antibiotic susceptibility across the 50,000-generation samples for all of the antibiotics except streptomycin, which showed no changes in any of the evolved lines (and hence no correlation with any other antibiotic). For the other 91 antibiotic pairs (14 × 13/2), we calculated the correlation coefficient for the changes in susceptibility using the log_2_-transformed difference in median MIC values between an evolved clone and the common ancestor. Figure 2 shows the resulting correlation matrix, which supports several points. First, there are many strong positive correlations across the antibiotics in the evolved changes in susceptibility. A total of 49 of the 91 correlations are positive and statistically significant at a *P *value of *<*0.1. Of these cases, 14 are significant at a *P *value of *<*0.01, including 5 cases at a *P *value of *<*0.001.

Second, many of the strong positive correlations reflect shared cellular targets of the antibiotics (Table 1), as indicated by the hierarchical clustering based on the correlation coefficients (Fig. 2). In particular, the six beta-lactam antibiotics tested in this study (CTX, CRO, TIC, AMC, AMP, and IPM), all of which inhibit cell wall synthesis, show positive correlations, with those between the cephalosporins (CTX and CRO) and the carboxypenicillin (TIC) being exceptionally strong. The responses of the evolved clones to the polymyxin CST, which disrupts both the inner and outer cell membranes, are also strongly correlated with the beta-lactams. In addition, the responses to the two quinolones used in this study (LVX and CIP), which inhibit DNA synthesis, are strongly correlated with one another.

Third, there are some exceptions to these patterns. For example, the responses of the evolved lines to TET and SXT, which inhibit synthesis of proteins and folic acid, respectively, are strongly positively correlated with responses to the quinolones, which inhibit DNA synthesis. SXT has also been reported to inhibit DNA synthesis indirectly (32), which might explain those correlated responses. Also, some correlations are evident even in those cases where the bacteria tended to become more resistant, not more susceptible. The evolved bacteria tend to be more resistant than the ancestor to CHL, which inhibits protein synthesis, yet these changes are positively correlated with the responses to the beta-lactams. Similarly, the responses of the evolved clones to CST, which include multiple cases of both increased resistance and increased susceptibility, are also correlated with the greater resistance to the beta-lactams.

### No compelling relationship between increased susceptibility and hypermutability.

During the LTEE, six of the 12 populations evolved hypermutability owing to mutations that affected either mismatch repair or the removal of oxidized nucleotides (19, 33, 34). The resulting point mutation rates were ∼100-fold higher than the ancestral rate, although most of the hypermutable lineages later evolved lower rates as the result of compensatory mutations or reversions (19, 35, 36). Previous work showed that hypermutable lineages tend to lose unused catabolic functions at a higher rate than other lineages (22, 25, 37), and it is possible that unused resistance traits would also decay faster in the hypermutable lines. We calculated the average of the log_2_-transformed changes in MICs across the 15 antibiotics for each of the 50,000-generation clones. We then compared averages for the six hypermutable and six other populations (Table 2), but there is no significant difference (*P = *0.3634, one-tailed *t* test with 10 degrees of freedom). This finding implies that the tendency to increased antibiotic susceptibility of the evolved bacteria does not result simply from the accumulation of mutations in unused genes. Instead, the greater susceptibility appears to reflect pleiotropic side effects of beneficial mutations that accumulated to a similar extent in the lineages that became hypermutable and those that retained the low ancestral mutation rate (18, 22).

**TABLE 2:** Bacterial strains used in this study^*a*^

Strain identifier	LTEE population	Generation
REL606	Ancestral strain	0
REL1158A	Ara+1	2,000
REL1159A	Ara+2	2,000
REL1160A	Ara+3	2,000
REL1161A	Ara+4	2,000
REL1162A	Ara+5	2,000
REL1163A	Ara+6	2,000
REL1164A	Ara–1	2,000
REL1165A	Ara–2	2,000
REL1166A	Ara–3	2,000
REL1167A	Ara–4	2,000
REL1168A	Ara–5	2,000
REL1169A	Ara–6	2,000
REL11392	Ara+1	50,000
REL11342	Ara+2	50,000
REL11345^*b*^	Ara+3	50,000
REL11348	Ara+4	50,000
REL11367	Ara+5	50,000
REL11370^*b*^	Ara+6	50,000
REL11330^*b*^	Ara–1	50,000
REL11335^*b*^	Ara–2	50,000
REL11364^*b*^	Ara–3	50,000
REL11336^*b*^	Ara–4	50,000
REL11339	Ara–5	50,000
REL11389	Ara–6	50,000

a All strains derive from *E. coli* B REL606 or REL607, an Ara+ mutant of REL606 (17, 19).

b These clones are from populations that were hypermutable for much of the time during the LTEE.

## DISCUSSION

Our study addresses two related questions, one of a fundamental nature and the other with potential biomedical implications. First, what happens to an organism’s capacity to deal with environmental stresses when it evolves for a long period without encountering those stresses? Second, does antibiotic resistance, including intrinsic resistance not associated with a known history of selection for resistance, tend to decay over time in the absence of exposure to antibiotics? To address these questions, we quantified changes in resistance profiles of 12 E. coli lineages after they had evolved in the absence of antibiotics for 50,000 generations.

On balance, we observed a clear tendency for antibiotic resistance to decline in the evolved clones, although there were some exceptions. Across all 15 antibiotics tested and both 2,000- and 50,000-generation evolved clones, and using a stringent criterion that all three replicate assays of an evolved clone yielded MICs that were lower or higher than any of the three assays for the ancestor, there were 10 times as many cases in which an evolved clone was more sensitive than the ancestor, as there were cases where an evolved clone was more resistant.

Two distinct population-genetic processes could, in principle, account for the overall trend toward lower resistance (22). One mechanism is called antagonistic pleiotropy (AP). Here, it refers to mutations that are beneficial in one environment (e.g., the absence of antibiotic) but which are maladaptive in some other environment (e.g., the presence of antibiotic). The other mechanism is often called mutation accumulation (MA). It concerns mutations that accumulate by random drift (including hitchhiking with beneficial mutations), usually in genes that are said to experience “relaxed selection” because their products and functions are not used in the current environment but which are valuable under other conditions. It is often difficult to distinguish between these processes, which are not mutually exclusive and so may act in concert. Nonetheless, in the present study, two findings point to AP as being the more important process with respect to the evolved tendency toward lower antibiotic resistance.

First, most of the reductions in antibiotic resistance occurred during the first 2,000 generations of the LTEE, with only modest further reductions by 50,000 generations. This pattern is similar to the increases in fitness, which occurred at a much higher rate early in the experiment than later (18, 38, 39). Also, most mutations that rose to high frequency during the early generations of the LTEE were beneficial “drivers,” whereas the proportion of effectively neutral “passengers” increased over time (19, 34, 36). The fact that the reductions in resistance roughly follow the trajectories for the increase in fitness and the rise of beneficial mutations is what one expects under the AP hypothesis (22). In contrast, if the declines in resistance resulted primarily from relaxed selection on the relevant gene functions, then under the MA hypothesis, the responsible mutations should accumulate and resistance should decay at a more or less constant rate.

Second, one reason even neutral mutations might not accumulate at a constant rate is if the mutation rate itself changes. In fact, such changes have occurred repeatedly during the LTEE, with six of the 12 populations having evolved hypermutable phenotypes as a consequence of mutations that affect DNA repair and metabolism (19, 33–36). The effects are large, with point mutation rates increasing by roughly two orders of magnitude in the hypermutable lineages, though many of them subsequently evolved partial compensatory mutations or reversions. Yet, we see no evidence that the LTEE populations that evolved hypermutability show any greater tendency toward increased antibiotic susceptibility. Under the MA hypothesis, however, we would expect to observe much greater losses of resistance in those lineages, owing to the accumulation of many more mutations in genes that confer resistance without adversely affecting fitness under the antibiotic-free conditions of the LTEE (22, 25).

Given the absence of a significant effect of extreme hypermutability along with the much higher average rate of decay in resistance early in the LTEE than later, we conclude that the declines in intrinsic resistance have resulted largely from pleiotropic side effects of beneficial mutations that were favored in the LTEE regime. Future work might identify the specific mutations that contributed to the declining resistance levels. At present, we simply highlight several genes that showed parallel (i.e., repeated) changes during the first 2,000 generations of the LTEE, which are therefore candidates in this regard, as follows: *mrdAB* and *mreBCD*, which are involved in peptidoglycan synthesis and cell wall structuring; *pykF*, which encodes a pyruvate kinase; *spoT*, which encodes a key protein in the stringent response; and *topA*, which encodes a topoisomerase involved in DNA supercoiling (19, 34, 36). The genes involved in peptidoglycan synthesis seem especially promising candidates for the increased susceptibility to the β-lactams (Table 1). More generally, many of the genes that have evolved most often during the LTEE have pervasive regulatory effects (24, 28, 40, 41). In that regard, a recent study showed that changes in the expression levels of many genes, including some not known to encode drug targets, were sufficient to affect the susceptibility of E. coli to antibiotics (42).

In this Discussion, we have emphasized the changes in intrinsic resistance. We do so because the low MICs of the LTEE ancestor are typical of antibiotic-sensitive strains of E. coli. Also, that ancestor has no known history of exposure to most antibiotics. The ancestor is a derivative of E. coli B, which was already in the laboratory by early in the 20th century (20), well before the use of antibiotics to treat disease. Moreover, there was no history of intentional selection for antibiotic resistance in the laboratory, with one exception, a progenitor to the LTEE ancestor that was selected for resistance to streptomycin in about 1966. Streptomycin targets the 30S subunit of the ribosome, and a mutation in the *rpsL* gene conferred resistance (11, 21).

Streptomycin is the only antibiotic where we saw no changes in susceptibility in any of the LTEE clones even after 50,000 generations. This retention of high-level resistance is perhaps surprising because many studies have shown that antibiotic resistance is often costly to bacteria in terms of growth and competitive fitness in the absence of antibiotics (4–9). Indeed, such costs have been observed for mutations that confer streptomycin resistance, including mutations in a codon immediately adjacent to the *rpsL* mutation in the LTEE ancestor (11, 31). However, a number of studies have found that compensatory mutations can often ameliorate these costs while maintaining resistance (10–16, 43). In fact, allelic replacement experiments indicate that a progenitor to the LTEE ancestor had already acquired a compensatory mutation (one that made reversion to streptomycin sensitivity costly) prior to the start of the LTEE (11). That finding thus explains why high-level streptomycin resistance persisted in all 12 LTEE populations even after 50,000 generations without antibiotic exposure.

The retention of streptomycin resistance notwithstanding, our results reveal a clear tendency for the erosion of low-level intrinsic resistance to antibiotics over time. The reasons for this trend are not known, in part because the evolutionary origins and physiological bases of low-level intrinsic resistance are poorly understood. The notion of intrinsic resistance is usually applied to those cases in which bacteria have a high level of resistance to certain antibiotics owing, for example, to efflux pumps or the impermeability of the cell envelope to those drugs (3). Low-level intrinsic resistance, in contrast, has received much less attention (however, see, e.g., references 26 and 44–48). Our results with the LTEE lineages show that even low levels of intrinsic resistance can sometimes go still lower, and sometimes much lower. This finding raises the question of whether low-level intrinsic resistance involves the same genes and functions as high-level intrinsic resistance (but with those functions expressed at lower levels) or whether it involves different mechanisms that have not yet been identified. It also raises the question of whether low-level intrinsic resistance evolved as an adaptation to deal with specific environmental challenges (e.g., antimicrobial molecules produced by competitors or hosts) or is coincidental to core metabolic functions. These questions remain open for further investigation.

It is also interesting to compare and contrast our results with those from Knöppel et al. (26). In that study, the authors propagated a total of 52 populations of either E. coli strain MG1655 or Salmonella enterica serovar Typhimurium LT2 for 500 to 1,000 generations in several media without antibiotics, and they assessed changes in resistance to 10 antibiotics. Of the 520 resulting comparisons in that study, the evolved cells became more susceptible in only 3 cases (0.6%), whereas they became more resistant in 18 cases (3.5%). In contrast, we saw increased susceptibility in 50.6% and 61.1% of our test combinations after 2,000 and 50,000 generations, respectively, while 13.3% and 11.7% exhibited greater resistance at those same time points. (These proportions are based on median MICs; they would be somewhat lower if we used the more stringent criteria that we also presented in the Results.) It is not possible to say definitively what is responsible for the differences between the two studies. One potentially relevant factor is their length, with the longer duration of our evolution experiment allowing more mutations to accumulate, each of which could impact antibiotic susceptibility. In this regard, it is interesting that although Knöppel et al. observed more cases of increased resistance than increased susceptibility, our study found a higher proportion of total cases in which increased resistance evolved. Thus, both studies indicate that bacteria sometimes evolve increased antibiotic resistance even without exposure to drugs. These two studies also differ in other respects, including the particular bacterial strains, media, and antibiotics employed. In this respect, one of the more striking differences is in the responses to rifampin. Knöppel et al. reported large increases in resistance to this antibiotic in several E. coli lineages, whereas in our study, none of the lines had increased resistance, and most of them became more susceptible. Differences in genetic background could well matter in this case (42, 49), as could differences in the culture media and other environmental conditions during the evolution experiment.

In summary, we examined changes in antibiotic-resistance profiles for 12 lineages of E. coli that evolved for 50,000 generations in antibiotic-free medium. We observed a clear trend toward increased susceptibility, even though the ancestor was already highly susceptible to all but one of the 15 antibiotics in this study. Most of the increases in susceptibility arose during the first 2,000 generations, a period when adaptation to the experimental environment was most rapid, and lineages that evolved hypermutability showed no greater tendency to become more susceptible. These patterns suggest that the increases in susceptibility were side effects of beneficial mutations in the experimental environment, rather than resulting from the decay of unused genes and functions. Against these overall trends, however, there were some exceptions, including small increases in resistance of some clones to a few antibiotics and the retention of high-level resistance to streptomycin.

## MATERIALS AND METHODS

### Bacterial strains.

The strains used in this study come from the E. coli long-term evolution experiment (LTEE) (17, 18, 36). In the LTEE, 12 populations, labeled Ara–1 to Ara–6 and Ara+1 to Ara+6, all derive from a common ancestral strain called REL606 (17, 20). They have been propagated since 1988 by daily 1:100 transfers in Davis minimal medium with 25 µg/ml glucose as the limiting resource (17). Samples are periodically collected from the evolving populations and stored at –80°C. In this study, we analyzed clones sampled from each population after 2,000 and 50,000 generations of the LTEE; the complete genomes of these clones were previously sequenced and mutations identified by comparison with the ancestral genome (19). Table 2 shows the strains used in this study.

### Culture conditions and measurement of MICs.

All experiments were performed at 37°C. Bacterial strains were first grown for 24 h in Davis minimal medium supplemented with 1 mg/ml glucose. These precultures were then diluted 1:100 in MH1000 medium, which consists of Mueller-Hinton broth (AES, Bruz, France) supplemented with 1 mg/ml glucose, 0.1 mg/ml MgSO_4_, and 0.01 mg/ml thiamine.

Table 1 shows the 15 antibiotics used in this study. We prepared stock solutions of each, according to the manufacturers’ instructions, which were then stored at –80°C. MICs were determined using a microdilution method in the MH1000 medium, as recommended by the CLSI (Clinical and Laboratory Standards Institute, Wayne, PA, USA, 2009). For each antibiotic, 50 µl of 2-fold serial dilutions in MH1000 were distributed per well in 96-well microtiter plates (Becton, Dickinson). An equal volume of a suspension containing ∼10^6^ cells/ml in MH1000 was added to each well, and the plates were incubated for 24 h with agitation at 120 rpm. The lowest antibiotic concentration that inhibited bacterial growth was recorded as the MIC. This inhibition was defined as no observable growth compared to an antibiotic-free control, and it was measured by eye. All experiments were performed in triplicate. For each experiment, we also performed in parallel a positive growth control (bacteria without any added antibiotic) and a negative control (MH1000 medium with the lowest antibiotic concentration tested, and without bacteria). We also included E. coli strain ATCC 25922 (Centre de Ressources Biologiques de l’Institut Pasteur, Paris, France) as an additional control for the determination of MICs.

### Data analyses.

As stated above, the MIC values were measured for each strain with 3-fold replication. Each value was transformed by taking its base-2 logarithm, which reflects the fact that the concentrations of antibiotics were tested across a series of 2-fold dilutions. The resulting values present a statistical challenge because they are discrete rather than continuous, and because the replicate assays often produced numerical ties, leading to imprecise and lumpy estimates of variance. Therefore, our analyses focus on broad patterns across the evolved samples and antibiotics rather than the response of any particular strain to a given antibiotic. We report the specific statistical test we performed to support a given conclusion with the corresponding results described above. We generally computed one-tailed *P* values to reflect our expectation that the bacteria would evolve to be more susceptible (lower MICs), owing to the absence of antibiotic exposure during the LTEE.
